# Mitigating the Risk of COVID-19 Deaths in Cardiovascular Disease Patients in Africa Resource Poor Communities

**DOI:** 10.3389/fcvm.2021.626115

**Published:** 2021-02-16

**Authors:** Ihunanya Chinyere Okpara, Efosa Kenneth Oghagbon

**Affiliations:** ^1^Cardiology Unit, Department of Internal Medicine, Benue State University Teaching Hospital, Makurdi, Nigeria; ^2^Department of Chemical Pathology, Benue State University Teaching Hospital, Makurdi, Nigeria

**Keywords:** COVID-19 deaths, cardiovascular disease, levels of prevention, Africa, resource poor communities

## Abstract

The novel coronavirus disease 2019 (Covid-19) pandemic has affected millions of patients in almost all countries with over one million cases recorded in Africa where it is a major health challenge. Covid-19 is known to have significant implications for those with pre-existing cardiovascular disease (CVD) and their cardiologists. Patients with pre-existing CVD are at increased risk of morbidity and mortality from Covid-19 due to associated direct and indirect life threatening cardiovascular (CV) complications. Mitigating the risk of such Covid-19 deaths in resource poor communities requires the institution of preventive measures at the primary, secondary and tertiary levels of preventive phenomenon with emphasis at the first two levels. General preventive measures, screening and monitoring of CVD patients for complications and modification of drug treatment and other treatment methods will need to be implemented. Health policy makers and manager should provide required training and retraining of CV health care workers managing Covid-19 patients with CVD, provision of health education, personal protective equipment (PPE), and diagnostic kits.

## Introduction

The novel corona virus disease referred to as Covid-19 was identified in December 2019 in Wuhan, China. It is caused by Severe Acute Respiratory Syndrome Coronavirus 2 (SARS-CoV-2) (1, 2) and the disease has since spread all over the world including resource poor communities in Africa (3).

Given the rapid spread of the virus in early 2020, the disease was declared a pandemic by the World Health Organization (WHO) on March 11, 2020 (2). Within a short time there was a litany of literature on the disease with physicians in all specialties expected to be aware of the impact of this disease in their respective clinical care areas and the medical community at large (4).

In Africa, the spread of Covid-19 was feared for so many reasons (5): Firstly, large and densely populated areas and townships with widespread poverty and high migration make such places vulnerable to airborne pandemics. Secondly, existing epidemics of human immunodeficiency virus (HIV), tuberculosis (TB), and malaria were thought to make Covid-19 more severe and thus lead to increased morbidity and mortality. Lastly, the high prevalence of non-communicable diseases in Africa such as hypertension, CVD and diabetes which are known risk factors for severe cases of Covid-19 portends a poor outcome (3), and this is of concern to the index authors.

The clinical presentation of this disease ranges from asymptomatic to mild, severe, and critical cases. Its symptoms which are similar to common viral and parasitic infections in sub-Saharan Africa include fever, cough, dyspnoea, myalgias, fatigue, and diarrhea. In severe and critical cases, it presents with pneumonia, acute respiratory distress syndrome (ARDS), cardiogenic, and septic shock. Over time, it was shown that elderly populations with pre-existing medical comorbidities are most vulnerable to severe disease (5–7).

In sub-Saharan Africa, the high prevalence of CVD and their relationship to the disease means cardiologists will be actively engaged in the management of Covid-19 patients. Aside Covid-19 infection being associated with CV complications, infected individuals with pre-existing CVD have elevated risk of severe disease and worse outcomes (8–10). Additionally, therapeutics for Covid-19 have potential adverse CV effects due to significant drug-drug interactions with regular CV medications. Finally, CVD drugs may interfere with the pathophysiology of Covid-19 especially with viral relationship to ACE2 receptors (11, 12).

The management of severe Covid-19 cases in patients with CVD and other high risk conditions is costly in resource poor countries of Africa, thus the need to activate primary and secondary levels of prevention. Presently, Covid-19 mortality in African countries are not as high as expected (5, 13). This is due to many factors including the implementation of primary and secondary preventive measures.

Hence, this review aim to discuss the need to mitigate Covid-19 deaths in CVD patients in resource poor countries of Africa, and the measures to be put in place toward realizing this goal.

## Virology of SARS-CoV-2, Epidemiology and Pathophysiology of Covid-19

The SARS-CoV-2 virus belongs to the family of Coronaviridae which are enveloped viruses with non-segmented, single stranded, positive-sense ribonucleic acid (RNA) genome (14). A number of the SARS—related coronaviruses have been found in bats, thus suggesting they may constitute the zoonotic host for SARS-CoV-2, especially given that the viral genome is 96.2% identical to a bat coronavirus (15). Typical of corona viruses such as SARS and the Middle East Respiratory syndrome virus (MERS-CoV), they commonly cause respiratory illnesses which are the predominant manifestation of Covid-19 disease. The infectivity of Covid-19 is greater than that of influenza with an estimated R_0_ value of 2.28 (16). Similarly, death rate associated with Covid-19 is higher compared to <0.1% estimated recently for influenza by WHO, though it may be higher for the elderly, persons with comorbidities and persons in low resource settings (17). However, earlier coronaviruses infections such as SARS-CoV epidemic and MERS-CoV, had higher case fatality rates of 9.6 and 34.4%, respectively (18). Covid-19 disease however has spread more widely to affect larger populations and places than previous coronaviruses outbreaks (18, 19).

Since December 2019, Covid-19 disease has spread to all corners of the globe affecting over 37 million persons with more than 1 million deaths as of 11th October, 2020 (20) in over 100 countries across the world. Africa as of the same date has over 1 million cases with the highest number of 690,896 cases in South Africa and lowest of 414 in Eritrea. Nigeria with 60,103 cases of confirmed Covid-19 is the highest in West Africa sub-region (20). The crude case-fatality rate which was 3.8% in the USA in March 2020 (21, 22) fell to 2.8% in October, in same month it is 1.8% in Nigeria (20).

The clinical cases can either be asymptomatic or mild in a large proportion of patients and severe in a smaller portion (18). In China it was found that 81.4% of cases were mild requiring only symptomatic treatment and isolation, with severe disease in 13.9% of cases that needed supplemental oxygen therapy, and critical in 4.7% requiring intensive care unit (ICU) treatment including mechanical ventilation (22).

Studies show that SARS-CoV-2 as well as other coronaviruses use angiotensin-converting enzyme 2 (ACE2) protein; a homolog of ACE1 (9) for cell entry. ACE2 which is a type 1 integral membrane protein is highly expressed in lung alveolar cells and may expose humans to increased viral entry (15). After ligand binding, SARS-CoV-2 enters cells via receptor-mediated endocytosis in a manner akin to entry of HIV viruses in to body cells (23). The viral take-over of ACE2 receptors in Covid-19 infection deregulates lung protective pathway occasioned by uninfected receptors, thus contributing to viral pathogenicity (24). ACE2 is found primarily in the lower respiratory tract, rather than the upper airways (10). This distribution can explain the few upper respiratory tract symptoms typical of flu and why Covid-19 is not just a common cold (10, 17).

Clinicians are concerned about a possible link between SARS-CoV-2 and angiotensin 2 receptor blockers (ARBs) which could increase chances of adverse effects of the disease in CVD patients on this class of antihypertensives (17), Hence it is important that doctors understand clinical and preventive measures to reduce morbidity and mortality from Covid-19 among CVD patients.

## Reasons to Mitigate the Risk of Covid-19 Deaths in CVD Patients

Several reasons portend the need to mitigate the risk of Covid-19 deaths in CVD patients (Table 1) details of which are given below.

**Table 1:** Reasons to mitigate the risk of Covid-19 deaths in CVD patients.

1.	Association between pre-existing CVD and severe Covid-19 disease.
2.	Life threatening CV complications are seen in Covid-19 patients with or without pre-existing CVD.
3.	Significant drug interactions exist between CVD medications and therapies under investigation for Covid-19.

### Association Between Pre-existing CVD and Severe COVID-19 Disease

Different studies show the association between pre-existing CVD and severe Covid-19 disease. A meta-analysis of seriously ill Covid-19 patients found the prevalence of hypertension, cerebrovascular disease and diabetes to be 17.1, 16.4, and 9.7%, respectively, among them (8). The overall case fatality rate in Covid-19 patients is commonly <3% (18), but this increases to 10.5% in those with CVD, 7.3% in diabetes, and 6.3% in hypertensives (18).Similar findings showing more adverse events in CVD patients with Covid-19, have been reported in other investigations, whether in China, Europe, or sub-Saharan Africa (13, 25). In Ghana, the highest number of deaths occurred in Covid-19 patients with pre-existing hypertension and diabetes (13). This number will go up as more people become seriously ill with Covid-19 in the sub-region due to inadequate facilities and personnel.

Aside hypertension, other factors associated with increased deaths are age, diabetes, and hyperlipidemia. Age is a risk factor for hypertension, obesity, glucose intolerance, and reduced immunity (25–27); which are associated with increased risk of severe Covid-19 disease. Diabetes and hyperlipidemia causes dysregulation of the immune system in addition to deterioration of vascular integrity (27, 28). Thus, prevalent CVD may be a marker of accelerated immunologic aging/dysregulation and relate indirectly to Covid-19 prognosis. Other possible risk factors for severe disease in low income countries of sub-Saharan Africa include HIV, TB, Chronic Obstructive Pulmonary disease (COPD), Rheumatic Heart Disease (RHD), and cardiomyopathies (29).

### Cardiovascular Complications of Covid-19 in Patients With or Without Pre-existing CVD

Several investigations suggest SARS-CoV-2 infection is associated with life threatening CV complications in those with or without pre-existing CVD (10, 18). The CV complications includes myocarditis, acute coronary syndromes, arrhythmias, heart failure, cardiogenic shock, and venous thromboembolism. The recognition of these complications must be possible in health facilities in Africa for improved survival of cardiac patients.

#### Myocarditis and Acute Coronary Syndromes

Myocardial injury is increased in patients with myocarditis and acute coronary syndrome as a results of ARDS and severe Covid-19 (10, 30, 31). Elevated serum troponin levels are seen in many Covid-19 patients, with significant differences noted in survivors and those who succumbed to the viral disease (32). Some authors found that mean cardiac troponin I levels was significantly higher in severe Covid-19-illness compared to non-severe disease (33).

Increased levels of troponin T (TnT) has been found to be associated with Covid-19 disease, especially in those with pre-existing CVD. Of note, the highest mortality rates were observed in those with elevated TnT levels whether due to Covid-19 or prior CVD.

Other markers of acute cardiac injury in Covid-19 patients are abnormal electrocardiographic and echocardiographic findings in patients.

#### Cardiac Arrhythmia and Cardiac Arrest

The arrhythmias observed in severe Covid-19 infections are cause for concern as it is a significant contributor to adverse outcomes (23). Arrhythmogenesis appears to be a feature of coronaviruses as these have been reported in SARS and MERS patients. The different forms of dysarrhythmias in coronaviruses infections include branch block, atrial fibrillation, premature beats, QT interval elongation, and even sudden cardiac death (34). In hospitalized Covid-19 patients, cardiac arrhythmias were noted in 16.7% of patients in a Chinese cohort especially in those in ICU (6). Up to 60% of fatal cases of Covid-19 have arrhythmias and in some patients the cardiac arrhythmias are independently associated with in-hospital mortality (35). This is more so as African Americans have been found to have genetic susceptibility for Covid-19 associated sudden cardiac death (36). It is advised that new onset of malignant tachyarrhythmias in the setting of troponin elevation should raise the suspicion of underlying myocarditis or acute coronary syndrome and potential arrhythmias (32, 37).

Arrhythmias should be considered a major complication of Covid-19 and be watched out for when medications are being considered in resource poor settings.

#### Cardiomyopathy and Heart Failure

Heart failure was reported in 23.0% of patients with Covid-19 presentations (10). Whether heart failure is most commonly due to exacerbations of pre-existing left ventricular (LV) dysfunction or new cardiomyopathy is unclear (38). Right heart failure and associated pulmonary hypertension should be considered, in particular in the context of severe parenchymal lung disease and ARDS, which are common findings in severe Covid-19 disease.

#### Cardiogenic and Mixed Shock

The appearance of ground glass opacities in severe Covid-19 patient similar to that in ARDS on chest imaging (39) should be distinguished from that of coexisting cardiogenic pulmonary oedema. A possibility of *in situ* cardiogenic or mixed cardiac plus primary pulmonary causes of respiratory manifestations in Covid-19 (mixed presentation), should be considered in clinical assessment of patients.

#### Venous Thromboembolic Disease

Patients with Covid-19 are at increased risk of venous thromboembolism and this is said to be as high as 31% in critically ill subjects (40). Studies suggest abnormal coagulation parameters like D-dimers are very useful in the diagnosis (41). In various places, elevated D-dimer levels (>1 g/l) are strongly associated with in-hospital death, even after multivariable adjustment (10, 41). The elevation of D-dimers and FDP (fibrin degradation products) are synonymous with poor survival in severely ill Covid-19 patients as this may indicate presence of disseminated intravascular coagulation (DIC) (41).

### Drug Therapy and Covid-19: Interactions and Cardiovascular Complications

There are currently no specific effective therapies for Covid-19. However, it is worthy to note that significant drug interactions exist between CVD medications and therapies under investigation for Covid-19 (12, 42, 43) (Table 2).

**Table 2:** Drug therapy and Covid-19: interactions and cardiovascular complications.

1.	Therapies under investigation for Covid-19 may have significant drug-drug toxicity with CV medications.
2.	Therapies under investigation for Covid-19 have significant CV toxicities.
3.	Patient debilitation from severe Covid-19 may pose challenges in administering routine CV medications
4.	Drugs for patients with CVD could interfere with the pathophysiology of Covid-19

## Mitigating the Risk of Covid-19 Deaths in CVD Patients in Africa Resource Poor Communities

Efforts to reduce Covid-19 deaths in CVD patients should involve three levels of prevention; primary, secondary, and tertiary levels. Primary prevention measures are those are put in place before the onset of illness. Secondary prevention refers to measures that ensure early diagnosis and prompt treatment, before development of CV complications. The tertiary prevention strategy is aimed at rehabilitation following significant illness.

In resource poor communities in sub-Saharan Africa, emphasis should be on primary and secondary preventive measures due to unsustainable financial requirements for tertiary measures of prevention.

Control measures will vary between:

1. Patients with CVD without Covid-19.
2. Patients with coexistence of CVD and Covid-19.
3. CV health workers.

Adequate consideration should be given to patients in resource poor communities where other immunosuppressive conditions such as HIV and TB could coexist with Covid-19 and CVD. Lifestyle measures, drug treatment and method of treatment modifications, as well as availability of necessary protective and medical equipment will all be required. Health care workers are also at risk of infection and should be protected.

### Mitigating the Risk of Death in Patients With Pre-Existing CVD Without Covid-19

Measures to reduce the risk of death in resource poor settings should be emphasized at the primary and secondary levels of prevention for sustainability. Since disease transmission occurs most commonly via respiratory droplets and aerosols with the virus active on surfaces for several days (44), the recommendations are suggested for general prevention include:

- All aged CVD patients should be taught to avoid close contact by practicing social distancing of at least 2 m away. They should be trained in community and personal hygiene and this should be more so with the uneducated subjects.
- As much as possible, patients with known risk factors should avoid crowds, especially in door assembly. Possibly, very vulnerable subjects should practice voluntary isolation but be able to receive support from family members to prevent depressive events. This isolation is important for those in major congested cities in Africa (13).
- Everyone must reduce or avoid touching their eyes, nose, and mouth, when up and about in their location where surfaces may be contaminated (44).
- Subjects must wash their hands frequently under running water. The alternative is to use alcohol (65% w/v ethyl alcohol) based hand sanitizers for similar purpose.
- The use of face masks should be mandatory for CVD subjects in resource poor settings.
- Pseudo-telemedicine approaches such as use of internet based telephone consultations; these include WhatsApp and Facebook videos which are popular in Africa and can be used for patient consultations, during pandemics to reduce travels and social contacts at hospitals. This can help to promote viral containment (45).

### Mitigating the Risk of Death in Covid-19 Patients With Pre-existing CVD in Sub-Saharan Africa

The majorly secondary and feasible tertiary levels of prevention features are as follows:

- Screening for Covid-19 in all CVD patients for early diagnoses, especially when they are susceptible groups like health and other frontline workers, or those with immune compromise. This will enhance early diagnosis and closer monitoring for CV complications (10, 18) in resource poor communities.
- Screening of Covid-19 patients for CVD and CV complications—The American College of Cardiology (ACC) has recommended the establishment of protocol for diagnosis, triage, and isolation of Covid-19 patients with CVD or CV complications (46).
- Telemedicine and e-visits—as mentioned above, with the wide availability of cell phones in resource poor communities in sub-Saharan Africa consultations can be made by patients with specialty physicians without close contact.
- Clear and prompt understanding of the effects of the virus and hypertension therapy in relation to ACE inhibitors and ARB therapy in Covid-19 patients, should be given early to reduce clinician and patient confusion (47). All CVD patients should be encouraged to continue their home blood pressure monitoring and medical regimen (48).
- All drug—drug interactions with CV medications and direct CV toxicities should be avoided or reduced to the barest minimum, by retraining of clinicians and other healthcare workers.
- As a tertiary measure, nationwide training of health workers on mechanical ventilatory support and Advanced Cardiac Life Support (ALCS) and all citizens on cardiac and vocational rehabilitation post Covid-19, should be commenced pending availability of resources for full implementation.

### Recommendations for Healthcare Workers Managing CVD During Covid-19 Pandemic

- Ensure the use of provided PPE and in the right manner as recommended by WHO, CDC and China's CDC, namely: facemask, goggles, disposable, or re-useable gowns and gloves (49–52).
- Telemedicine and e-visits—this allows for triaging of patients and patient management while minimizing exposure of patients and health workers to potential infection.
- Health care practitioners must be conversant with antiviral agents approved or under investigation for the treatment of Covid-19 and their CV toxicities (53).
- Carefully managed rescheduling of elective procedures during the growth phase of the outbreak.
- Ensure hospital equipment such as echocardiography, scanners et cetera are cleaned with antiseptic agents before and after each use.
- When performing procedures that generates aerosol such as transesophageal echocardiography, endotracheal intubation, cardiopulmonary resuscitation, and bag mask ventilation, additional PPE may be required including controlled or powered air purifying respirators. Thorough infection control measures specific to the procedural cardiology specialty should be considered in light of Covid-19 outbreak.
- In the event of cardiac arrest, use of external mechanical chest compression devices would help to minimize direct contact with infected patients.
- The healthcare worker must self-report symptoms if present, and be excused from duty as health worker when symptomatic until tested and found negative.
- Overall, as CV health workers are on the front lines treating Covid-19 patients, all possible measures should be implemented to reduce the risk of exposure (54).

### Recommendations for Health Policy Officials and Manager

- Provision of infrastructure for e-visits and telemedicine where possible.
- Provision of sufficient PPEs for patient families and health care personnel.
- Improving patient and public education concerning Covid-19 infection.
- Provide adequate tests materials and personnel so that appropriate containment can be achieved.

## Conclusion

The Covid-19 pandemic has affected thousands of patients globally, but its impact on resource poor communities in sub-Saharan Africa constitutes a major international health challenge. Where as many CVD patients have not died because of the virus, but a significant number had poor outcomes because of fear of going to the hospitals, or because hospitals have shut out routine care in most resource poor environment. Mitigating the risk of death from this disease will involve training and retraining of health care workers and ensuring provision of primary, secondary and tertiary levels preventions. Efficient resources channeled to combat this pandemic by health policy makers and managers will go a long way to mitigate risk of death, if actions are taken early and in right measures.

## Data Availability Statement

The original contributions presented in the study are included in the article/supplementary material, further inquiries can be directed to the corresponding author.

## Author Contributions

IO carried out data collection and manuscript writing. EO was involved in manuscript writing and editing of manuscript. All authors contributed to the article and approved the submitted version.

## Conflict of Interest

The authors declare that the research was conducted in the absence of any commercial or financial relationships that could be construed as a potential conflict of interest.
